# Baicalin Attenuates Continuous Activation of *β*-Catenin Induced by Lipopolysaccharide (LPS) and Depression Complicated by Infertility in Male Rats

**DOI:** 10.1155/2022/2112359

**Published:** 2022-04-07

**Authors:** Rong Fan, Feng Dai, Chunhu Zhang, Lu Zhou, Xinjian Qiu, Yunhui Li

**Affiliations:** ^1^Department of Integrated Traditional Chinese and Western Medicine, Xiangya Hospital, Central South University, Changsha, China; ^2^Department of Emergency, Xiangya Hospital, Central South University, Changsha, Hunan, China; ^3^A Phase I Clinical Trial Center, Xiangya Hospital, Central South University, Changsha, China

## Abstract

**Background:**

Baicalin (BA) is a potential candidate drug to inhibit depressive behavior. However, the mechanism of BA's role on depression complicated with male infertility (DCMI) is still unclear. This study aimed to investigate the role of BA in alleviating inflammatory factor-induced DCMI by regulating *β*-catenin.

**Methods:**

Firstly, we performed sucrose preference test (SPT), open field test (OFT), tail suspension test (TST), and forced swim test (FST) in the chronic unpredictable mild stress (CUMS) + lipopolysaccharide (LPS) model rats to study the effect of BA on depressive behavior. The levels of neuropeptide Y (NPY), testosterone (*T*), and IL-1*β*, IL-6, TNF-*α*, IL-10, and IL-4 in the peripheral blood plasma of normal people, patients with depression, and patients with DCMI were measured. Then, the levels of IL-1*β*, IL-6, TNF-*α*, IL-10, IL-4, *β*-catenin in rat testis and peripheral blood and ANXA2, APP, SEMG1, and SEMG2 in seminal plasma proteins were examined. Moreover, the level of *β*-catenin in the testicular tissue was detected. LPS was used to treat Sertoli cells, and the level of *β*-catenin was detected. Finally, we evaluated the reproductive phenotype and sperm motility of rats.

**Results:**

BA (especially 100 mg/kg) could notably ameliorate depression-like behavior induced by CUMS + LPS. The levels of IL-4, IL-10, *T*, and NPY in depression patients, DCMI patients, and CUMS + LPS model rats elevated, while the levels of IL-1*β*, IL-6, and TNF-*α* were reduced. However, BA alleviated the changes in these factors. Moreover, BA alleviated male rat depression induced by CUMS + LPS. LPS upregulated *β*-catenin (NP) but could not adjust *β*-catenin (TP) level in rat Sertoli cells. BA relieved the symptoms of DCMI by regulating *β*-catenin. Furthermore, BA ameliorated the reproductive ability of depressed rats.

**Conclusion:**

BA modulated *β*-catenin in the relief of inflammatory factor-induced DCMI.

## 1. Introduction

Depression is the most common comorbid psychiatric disorder in the world population and in the primary health care [1]. The clinical characteristics of depression are low mood and anxiety, accompanied by internal and mental restlessness [2]. Long-term depression will affect the normal metabolism of the human body. To some extent, it will cause endocrine disorders, affect androgen secretion, hinder the normal movement of sperm, and lead to sterility [3], which is known as depression complicated with male infertility (DCMI). Recently, the relationship between depression and infertility treatment has been in the spotlight. Severe depression can reduce pregnancy rates during infertility treatment with assisted reproductive technology [4]. DCMI is a difficult problem in clinical medicine. Therefore, researchers in this field should continuously address this problem.

Sertoli cells were closely related to the differentiation of germ cells and the targets of follicle-stimulating hormone and testosterone (T), which regulate spermatogenesis [5]. Wnt signaling pathway is indispensable for normal testicular function [6]. *β*-catenin is a protein with dual functions in cells, which plays a role in intercellular adhesion and gene transcription through the canonical Wnt/*β*-catenin signaling pathway [7]. Li et al. reported that the Wnt/*β*-catenin signaling pathway was related to the proliferation of spermatogonia. Abnormal expression of inflammatory factors plays a role in the pathophysiology of depression [8–10]. At the same time, the Wnt/*β*-catenin signaling pathway is closely related to inflammation [11, 12]. Therefore, we speculate that the stimulation of Sertoli cells by inflammatory factors leads to the activation of the Wnt/*β*-catenin signaling pathway, which promotes the incomplete differentiation of Sertoli cells and the exhaustion of germ cells, leading to infertility.

Baicalin (BA) is a flavonoid compound extracted from the root of *Scutellaria baicalensis* without obvious side effects [13]. Previous studies have shown that BA has anti-inflammatory, antioxidant, and antidepressant effects. Dai et al. found that BA protected neurons from cell death and enhanced neural function after cerebral ischemia [14]. Furthermore, BA has been found to attenuate learning and memory impairments induced by global cerebral ischemia/reperfusion, abrogate depressive-like behaviors caused by chronic mild stress, and prevent neuronal apoptosis in chronic unpredictable mild stress (CUMS) rats [15, 16]. Therefore, BA may represent a potential drug candidate for patients with depression [17]. More and more research reveals that BA has a role in treating depression. Studies have shown that BA could inhibit the neurogenesis of the hippocampus in mice through the Wnt/*β*-catenin signaling pathway, thereby slowing down the depressive behavior of mice [18]. However, the effect of BA on male infertility has not been studied. Therefore, we hypothesized that BA could affect DCMI by modulating the Wnt/*β*-catenin signaling pathway.

Based on the above studies, to better understand the pathophysiology of depression complicated by infertility, we investigated the mechanism of DCMI in rodents exposed to CUMS and lipopolysaccharide (LPS) at different concentrations of BA.

## 2. Materials and Methods

### 2.1. Clinical Sample Collection and Processing

Sixty male patients were admitted from Xiangya Hospital of Central South University from October 2019 to June 2021, aged 20 to 42 years, including 30 cases of depression and 30 cases of DCMI. Thirty normal people came from social health volunteers. Peripheral blood of all subjects was collected. All subjects agreed to provide the information required for the experiment. The experiment was approved by the Medical Ethics Committee of Xiangya Hospital of Central South University (202108383).

### 2.2. Cell Culture and Processing

Rat Sertoli cells were purchased from Wuhan Procell Co., Ltd (Wuhan, China, CP-R160). Cells were cultured in Dulbecco's modified Eagle medium (DMEM) containing 10% fetal bovine serum and 1% penicillin/streptomycin in a constant temperature incubator at 37°C and 5% CO_2_. Cells were seeded in two Petri dishes. In one Petri dish, cells were cultured normally (control group), and in other Petri dish, cells were added with 10 *μ*g/mL LPS for 24 h (10 *μ*g/mL LPS group) [19]. After treatment, cells were stored at −20°C for later experiment.

### 2.3. Animal Modeling and Grouping

Forty adult C57BL/6 male rats (180–220 g) were purchased from Hunan SJA Laboratory Animal Co., Ltd. The rats were randomly housed in the cages under a 12 h light/dark cycle (lights on at 7:00 a.m.), 60% humidity, and temperature at 24 ± 1°C with free access to the water and food. All of the procedures were strictly performed according to the Provision and General Recommendation of the Chinese Experimental Animals Administration Legislation and were approved by Xiangya Hospital of Central South University (Approval No. 202108011).

We performed CUMS on all rats except for the control group [20, 21]. Nine different pressures (water shortage for 20 h, water shortage for 18 h, shroud tilted at 45°C for 17 h, overnight lighting, wet cage feeding for 21 h, swimming in 4°C water for 5 min, stay on a swing bed at 160 Hz for 30 min, tail kneading for 1 min, and fixing for 2 h) were randomly assigned to rats during 42 consecutive days of the day and night. On the 8th day after CUMS, rats were injected intraperitoneally with LPS (500 *μ*g/kg) every other day for two weeks. In the CUMS + LPS + BA (25, 50, and 100 mg/kg) group on the 22nd day after CUMS, rats received different concentrations of BA (25, 50, and 100 mg/kg) every other day for 21 days [22, 23]. The control and CUMS + LPS groups were injected with the same amount of saline as BA. The behavioral test was performed on the 43rd day. After the experiment, the rats were euthanized. The testes, epididymis, peripheral blood, and semen of all rats were collected.

### 2.4. Depressive Behavior Test

All rats were tested for depressive behavior at the same time. We used the sucrose preference test (SPT), open field test (OFT), tail suspension test (TST), and forced swim test (FST) to assess the degree of depression in rats. First, we performed SPT on rats [24]. Before the test, the rats were housed in the cage with sucrose water (1% (w/v)) and tap water (regular water) to acclimate for 24 h. After acclimatization, the rats were deprived of water and food for 12 h. Subsequently, we reared the rats individually and gave two bottles of sucrose water (1% (w/v)) and tap water for 12 h, alternating the positions of two bottles every 6 h alternately to eliminate the possibility of side or position preference. All SPT measurements were performed at night due to the influence of circadian rhythm on the drinking water of rats. The formula for calculating the sucrose preference (SP) value was as follows: SP (%) = sucrose intake (g)/(sucrose intake (g) + water intake (g)) × 100%.

Then, we performed OFT on the rats [25, 26]. To assess anxiety, we placed the rats individually in the center of an open device, which included a square wooden arena (50 cm × 50 cm × 40 cm), with the bottom divided into 25 black and white square lattices. The number of times each rat crossed the border of the small grid and entered the next grid within 6 min was recorded. The equipment was cleaned with ethanol after each test.

Next, we performed TST on the rats [27, 28]. The rear 1/3 of the tail of the rat was fixed with tape and hung on the bracket, with the head 15 cm away from the table. After acclimatization for 2 min, the immobility time of rats was counted in the next 4 min.

Finally, we performed FST on the rats [29, 30]. Briefly, each rat was placed in a glass cylinder (20 cm high, 14 cm diameter) filled with water (25 ± 2°C) to a height of 10 cm. The immobility time of rats after forced swimming for 6 min was recorded after 4 min.

### 2.5. Sterility Test

First, we evaluated the reproductive phenotype of rats. When the rats were grown to 6 weeks old, one male rat and four female rats with proven fertility were placed in the same cage for one week (T0). The mating plug (coagulated semen) of female rats was monitored daily as a sign of mating behavior. After one week, male rats proved to be fertile were grouped and fed separately according to the above animal experiments. The investigation was also performed according to the abovementioned experimental methods. In the third week (T3) of the experiment, each rat was kept in the same cage with four female rats for one week, and the mating plug of female rats was monitored daily. Similarly, in the 6th week (T6) of the experiment, each rat was caged with four female rats for one week, and the mating plug of female rats was monitored every day.

### 2.6. Sperm Motility Test

We put an epididymal tail into the 35 mm dishes containing 1.5 mL of bicarbonate buffered human fallopian tube fluid medium, which was supplemented with 3 mg/mL BSA and covered with liquid paraffin. After that, we used a tuberculin syringe with a 26G needle to quickly rupture the epididymis, and then, we gently squeezed the epididymal sperm out of the small tube. Next, we cultured the epididymis in the Petri dish, released sperm in an incubator at 37°C and 5% CO_2_ for 30 min, and then we removed the epididymal tissue immediately. The sperm suspension was diluted at 1 : 10 in a pre-balanced medium (37°C). A small amount of suspension was immediately absorbed, added to a preheated slide (37°C), and allowed to stand for 20 s. Then, we immediately used a microscope to evaluate sperm motility at 37°C blindly. At least 200 spermatozoa were counted in each sperm sample. Sperm motility data were recorded as the number of progressive motility sperm of the total number of sperm in the grid × 100.

### 2.7. Enzyme-Linked Immunosorbent Assay (ELISA)

Fresh peripheral blood of humans and rats were centrifuged, the plasma was taken, and the plasma samples were stored at −20°C or −80°C for later experiments. The thawed samples were centrifuged again and then tested. According to the manufacturer's instructions, we used Neuropeptide Y (NPY) ELISA Kit (Cusabio, CSB-E08168h/CSB-E08170m), Serum *T* ELISA Kit (Cusabio, CSB-E05099h/CSB-E05101m), IL-1*β* ELISA Kit (Cusabio, CSB-E08053h/CSB-E08054m), IL-6 ELISA Kit (Cusabio, CSB-E04638h/CSB-E04639m), TNF-*α* ELISA Kit (Cusabio, CSB-E04740h/CSB-E04639m), IL-10 ELISA Kit (Cusabio, CSB-E04593h/CSB-E04594m), and IL-4 ELISA Kit (Cusabio, CSB-E04633h/CSB-E04634m) to detect the levels of NPY, *T*, IL-1*β*, IL-6, TNF-*α*, IL-10, and IL-4 in human and rat peripheral blood plasma.

### 2.8. Western Blot (WB)

RIPA lysis buffer was applied to extract total protein and nucleoprotein from rat testis tissue. According to the BCA Protein Determination Kit for protein quantification of each group, the protein was separated on a 10% SDS-PAGE gel and then transferred to the PVDF membranes. The membranes were sealed with a 5% skimmed milk solution at room temperature for 2 h, combined with the primary antibodies IL-1*β* (ProteinTech, 16806-1-AP), IL-6 (Abcam, ab229381), TNF-*α* (ProteinTech, 17590-1-AP), IL-10 (Abcam, ab271261), IL-4 (ProteinTech, 66142-1-Ig), MIS (Abcam, ab229212), ANXA2 (ProteinTech, 11256-1-AP), APP (ProteinTech, 25524-1-AP), SEMG1 (Abcam, ab139405), SEMG2 (ThermoFisher, PA5-88785), and *β*-actin (ProteinTech, 51067-2-AP/60008-1-Ig), and incubated overnight at 4°C. *β*-actin was used as internal reference *l*. TBST was used to wash the membranes three times. Then, the membranes were incubated with the secondary antibodies HRP goat anti-rat IgG (ProteinTech, SA00001-1) or HRP goat anti-rabbit IgG (ProteinTech, SA00001-2) 90 min at room temperature. After using ECL to develop color exposure, the Odyssey Infrared Imaging System was performed to detect protein bands.

### 2.9. Immunohistochemistry (IHC)

The testicular tissue of rats was fixed with paraformaldehyde, then embedded in paraffin, and cut into 4 *μ*m thin slices. The slices were baked at 60°C for 12 h, dewaxed with xylene, and then rehydrated. The slices were immersed in 0.01 M citrate buffer (pH = 6.0) and heated to boiling in a microwave oven. After boiling for 23 min, the slices were cooled to room temperature. To inactivate the endogenous enzyme, 1% periodate was added into the slices for 10 min at room temperature. The slices and primary antibody *β*-catenin (BIOSS, 51067-2-ap) were incubated overnight at 4°C. Then, the slices were added (50–100*μ*L HRP goat F (ab) anti-rabbit IgG (Abcam, ab7171)) and incubated at 37°C for 30 min. Then, DAB developer working solution of 50–100 *μ*L was added to the slices and incubated at room temperature for 1–5 min. Hematoxylin was used to counterstain the sections for 5–10 min, and PBS was used to return to blue. We dehydrated the slices with various alcohol levels (60–100%) for 5 min. After removal, the slices were placed in xylene for 10 min twice and sealed with neutral gum. Finally, we obtained fluorescence images under a confocal microscope.

### 2.10. TUNEL

The preparation and hydration process of the slices were the same as before. 100 *μ*L proteinase K working solution was dropped on each slice and reacted at 37°C for 20 min. Then, each slice was dropped with 100 *μ*L 1 × equilibration buffer and incubated at room temperature for 10–30 min. Around the equilibrated area, an absorbent paper was used to wash off most of the 100 *μ*L 1 × equilibration buffer. Then, 50 *μ*L TdT incubation buffer was added to the slices. The slices were incubated at 37°C for 60 min and kept away from the light. Under the dark condition, DAPI working solution was used to stain cell nuclei at 37°C. After 10 min, we used buffered glycerol to seal the slices. The fluorescence microscope was applied to obtain fluorescence pictures.

### 2.11. Statistical Analysis

SPSS 17.0 software was applied for statistical analysis. Quantitative data were expressed as mean ± standard deviation (***±SD***). *t*-Test was used for comparison between two groups. After the one-way ANOVA, the Student–Newman–Keuls test was performed for comparison between multiple groups, and the rank-sum test was used for comparison between groups of data that did not conform to normal distribution. *P* ≤ 0.05 was considered statistically significant.

## 3. Results

### 3.1. BA Affected Depressive Behavior in Rats

**Figure 1:**
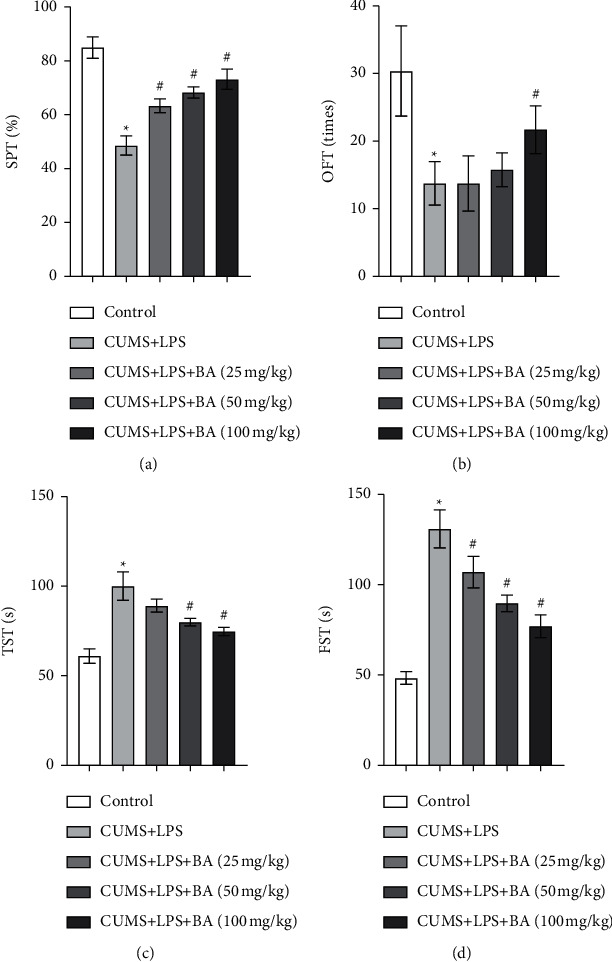
Effects of BA on depressive behavior in rats. (a) Sucrose preference test (SPT) in rats. (b) Open field test (OFT) in rats. (c) Tail suspension test (TST) in rats. (d) Forced swim test (FST) in rats. ^*∗*^*P* < 0.05 vs control group; # *P* < 0.05 vs. CUMS + LPS group.

The depressive behavior of rats in the control, CUMS + LPS, and CUMS + LPS + BA (25, 50, and 100 mg/kg) groups was tested, including SPT, OFT, TST, and FST. The results showed that the levels of SPT (Figure 1(a)) and OFT (Figure 1(b)) in the CUMS + LPS group were notably reduced compared with the control group. After the BA treatment, the levels of SPT and OFT in the CUMS + LPS and CUMS + LPS + BA (25, 50, and 100 mg/kg) groups showed significant regression, and the effect of treatment was more obvious with the increase in BA concentration. However, compared with the control group, the levels of TST (Figure 1(c)) and FST (Figure 1(d)) in the CUMS + LPS group were significantly elevated. After the BA treatment, the levels of TST and FST in the CUMS + LPS + BA (25, 50, and 100 mg/kg) groups reduced notably, and the therapeutic effect of 100 mg/kg BA on rats was the most obvious. It could be seen that BA improved the depressive behavior of rats induced by CUMS + LPS. The optimal therapeutic concentration of BA for CUMS + LPS rats was 100 mg/kg, and the therapeutic concentration was dependent.

### 3.2. BA Reduced Inflammation in DCMI

**Figure 2:**
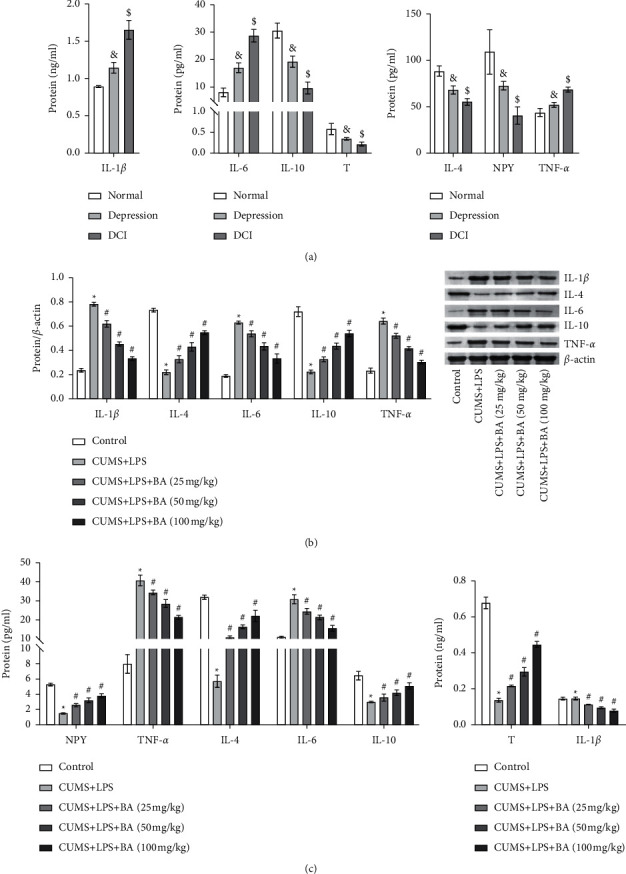
Expression of inflammatory factors in patients with DCMI. (a) The levels of plasma NPY, (t) IL-1*β*, IL-6, TNF-*α*, IL-10, and IL-4 in peripheral plasma were determined by ELISA. (b) WB was used to detect the levels of IL-1*β*, IL-4, IL-6, IL-10, and TNF-*α* in rat testicular tissue. (c) The serum levels of (T) IL-1*β*, IL-6, TNF-*α*, NPY, IL-10, and IL-4 were determined by ELISA. & *P* < 0.05 vs normal group; $ *P* < 0.05 vs. depression group; ^*∗*^*P* < 0.05 vs control group; #*P* < 0.05 vs. CUMS + LPS group.

To investigate the differences in the expression of inflammatory factors in the serum of normal person, patients with depression, and patients with DCMI, we performed ELISA to detect the levels of NPY, *T*, IL-1*β*, IL-6, TNF-*α*, IL-10, and IL-4 in the peripheral blood plasma. The results showed that the levels of NPY, *T*, IL-10, and IL-4 in the normal group, depression group, and DCMI group were from high to low (Figure 2(a)). However, the levels of IL-1*β*, IL-6, and TNF-*α* were just opposite to the expression trends of NPY, *T*, IL-10, and IL-4. It could be seen that there was inflammation in the patients with depression, and the inflammation was more serious in patients with DCMI. In addition, depression had led to lower NPY and *T* levels in the peripheral blood and caused endocrine disorders and immune disorders.

Next, we treated CUMS + LPS rats with different concentrations of BA and then detected the levels of inflammatory factors in the testis tissue. The WB results are shown in Figure 2(b). Compared with the CUMS + LPS group, BA treatment significantly inhibited the levels of IL-1*β*, IL-6, and TNF-*α* in the rat plasma and promoted the levels of IL-4 and IL-10. Finally, we used WB to detect the changes in inflammatory factors, NPY, and *T* in rat plasma. The results are shown in Figure 2(c). The levels of IL-1*β*, IL-6, and TNF-*α* were decreased in BA-treated rats. The levels of NPY, IL-10, IL-4, and *T* were increased. It could be seen that BA had a very obvious improvement on DCMI, which was in a concentration-dependent manner.

### 3.3. BA Reduced the Continuous Activation of *β*-Catenin Induced by Inflammation

**Figure 3:**
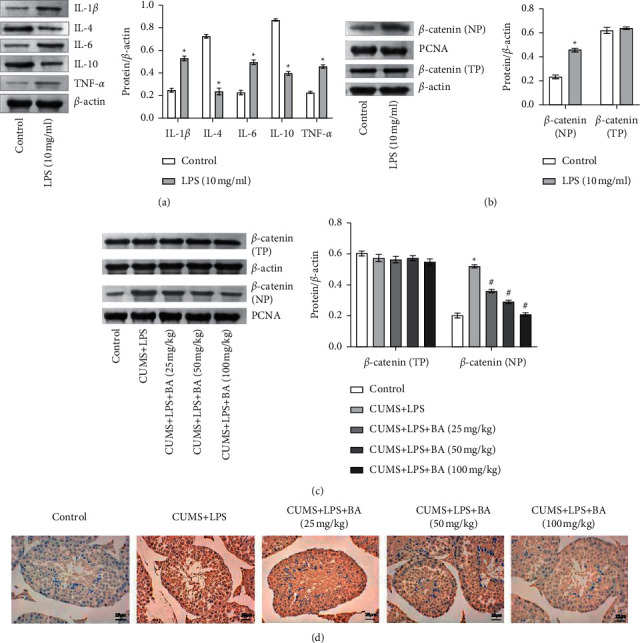
Inflammatory factors affected the expression of *β*-catenin in Sertoli cells. (a) The expression of IL-1*β*, IL-6, TNF-*α*, IL-10, and IL-4 in Sertoli cells was measured by WB. (b) The expression of *β*-catenin (TP) and *β*-catenin (NP) in Sertoli cells was detected by WB. (c) WB was applied to detect the expression of *β*-catenin (TP) and *β*-catenin (NP) in rat testicular tissue. (d) IHC was performed to detect the expression of *β*-catenin (NP) in rat testicular tissue. Scale bar = 25 *μ*m. ^*∗*^*P* < 0.05 vs control group.

To explore the effects of inflammatory factors on Sertoli cells, we treated Sertoli cells with LPS to induce cell inflammation. Subsequently, we used WB to detect the levels of IL-1*β*, IL-6, TNF-*α*, IL-10, and IL-4 in each group. The results showed that compared with the control group, the levels of IL-1*β*, IL-6, and TNF-*α* in the 10 *μ*g/mL LPS group were significantly upregulated, while the levels of IL-10 and IL-4 were downregulated (Figure 3(a)). Further, we explored the effects of LPS on *β*-catenin (nuclear protein, NP; total protein, TP) in Sertoli cells. The results showed that compared with the control group, the *β*-catenin (NP) level in the 10 *μ*g/mL LPS group was significantly upregulated, and the *β*-catenin (TP) level was not significantly different (Figure 3(b)). Immediately afterward, we continued to perform WB detection on the testicular tissues of rats. Compared with the control group, the level of *β*-catenin (NP) was increased in the CUMS + LPS group. After the BA treatment, the level of *β*-catenin (NP) decreased. However, *β*-catenin (TP) level was not significantly different in each group (Figure 3(c)). The IHC results are shown in Figure 3(d). Compared with the control group, the level of *β*-catenin (NP) was significantly inhibited after CUMS + LPS rats were treated with different concentrations of BA. The results indicated that the inflammatory response stimulated the level of *β*-catenin in the nucleus of the Sertoli cells but could not regulate the total protein level of *β*-catenin.

### 3.4. BA Alleviated the Decline of Reproductive Capacity in Depressed Rats Induced by Inflammation

**Figure 4:**
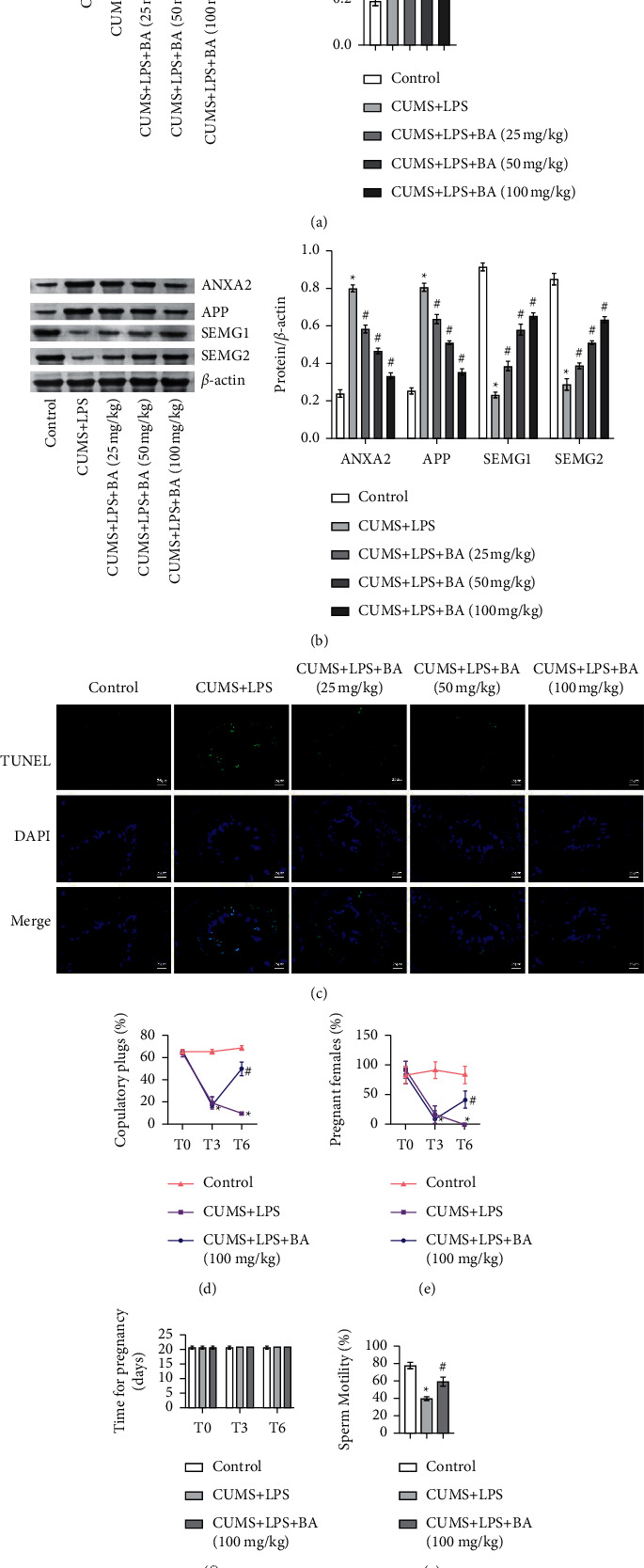
BA alleviated sterility by regulating *β*-catenin. (a) WB was used to measure the levels of MIS in rat testicular tissue. (b) WB was performed to detect the expression of ANXA2, APP, SEMG1, and SEMG2 in rat seminal plasma proteins. (c) TUNEL was applied to measure the apoptosis rate of germ cells. Scale bar = 25 *μ*m. (d) The number of mating plugs in each group. (e) The pregnancy rate of female rats mated with rats in each group. (f) Pregnancy time of rats in each group. (g) Sperm motility of rats in each group. ^*∗*^*P* < 0.05 vs control group; #*P* < 0.05 vs. CUMS + LPS group.

First, we detected the content of MIS (Müllerian) in rat testis tissue (Figure 4(a)). In the CUMS + LPS group, the content of MIS was increased compared with the control group, but after the BA treatment, the content of MIS decreased. Then, we used WB to detect the expression of ANXA2, APP, SEMG1, and SEMG2 in rat seminal plasma protein. The results showed that the expression of ANXA2 and APP in CUMS + LPS rats treated with BA was significantly inhibited, while the expression of SEMG1 and SEMG2 was promoted (Figure 4(b)). Next, we used TUNEL Apoptosis Detection Kit to detect cell apoptosis in rat testes. As shown in Figure 4(c), the number of apoptosis in the control group was the least. After treatment with BA, the apoptosis of testicular tissue cells in CUMS + LPS rats was reversed. To detect the effect of BA on the reproductive ability of each group, we evaluated the reproductive phenotype and sperm motility of the rats. The results showed that the number of mating plugs was increased after the BA treatment (Figure 4(d)). Moreover, the pregnancy rate of CUMS + LPS rats was decreased notably. After BA treatment, the pregnancy rate of rats was also increased (Figure 4(e)). There was no significant difference in the pregnancy time of each group (Figure 4(f)). After the BA treatment, the sperm motility of rats was also significantly improved (Figure 4(g)).

## 4. Discussion

In recent years, the anti-inflammatory effects of BA on the inflammatory diseases have been frequently studied. Many studies have shown that neuroinflammation plays a vital role in depression [31–34]. This research showed that BA showed the antidepressive and infertility effects by reducing neuroinflammation in CUMS + LPS model rats. The underlying mechanism was related to the regulation of *β*-catenin expression.

Previous clinical studies have revealed that elevated pro-inflammatory cytokines in the central and peripheral nerves might cause depressive behavior [35]. Anti-inflammatory cytokines IL-10 and IL-4 and pro-inflammatory cytokines IL-1*β*, IL-6, and TNF-*α* play a vital role in the occurrence and development of inflammation diseases [36, 37]. A survey showed that the level of *T* in patients with depression was lower than that in normal people [38]. Furthermore, both preclinical evidence and clinical evidence showed that the low level of NPY directly led to a variety of mental illnesses, including depression and related diseases [39–41]. Our study detected the levels of NPY, *T*, IL-1*β*, IL-6, TNF-*α*, IL-10, and IL-4 in the peripheral blood plasma of patients with DCMI. The results showed that the levels of IL-1*β*, IL-6, and TNF-*α* were notably elevated in the patients with DCMI, while the levels of NPY, *T*, IL-10, and IL-4 were notably reduced. Therefore, we speculated that patients with DCMI would suffer from endocrine disorders and immune disorders due to the long-term irresistible stress. This led to the low expression of anti-inflammatory factors IL-10 and IL-4 and the high expression of pro-inflammatory factors IL-1*β*, IL-6, and TNF-*α*. At the same time, the levels of serum *T* and NPY were higher than those of patients with depression. NPY and *T* might be the effective targets for treating depression and infertility. After that, we treated CUMS + LPS rats with different concentrations of BA. The results indicated that the inflammation in the rat serum and testis tissue has been relieved. At the same time, the levels of NPY and *T* have also been restored to a certain extent. It suggested that BA effectively alleviated the inflammation and immune disorders in depressed and infertile rats.

Studies have shown that BA could slow down neuroinflammatory cells apoptosis by regulating the Wnt/*β*-catenin signaling pathway [42–44]. Our results indicated that the expression trends of IL-1*β*, IL-6, TNF-*α*, IL-10, and IL-4 in Sertoli cells induced by LPS were consistent with the results of our clinical studies. The level of *β*-catenin (NP) was highly expressed, while the level of *β*-catenin (TP) was not significantly different. It was known that the entry of *β*-catenin into the nucleus was its activation symbol, which could enhance *β*-catenin transcription and increase the level of *β*-catenin. This indicated that LPS promoted the activation of *β*-catenin in Sertoli cells. At the same time, we used BA to treat CUMS + LPS rats. The results showed that after BA treatment, the level of *β*-catenin (NP) was decreased. This showed that BA alleviated depression in rats. Previous report showed that the seminal plasma proteins ANXA2 and APP were overexpressed in secondary infertility, and both SEMG1 and SEMG2 were underexpressed [45]. Pradeep S. Tanwar et al. showed that the expression of AMH/MIS (Müllerian) was a marker of immature Sertoli cells and was regulated by the transcription of *β*-catenin [46]. In our experiments, BA indeed repressed the expression of ANXA2 and APP in CUMS + LPS rats and promoted the expression of SEMG1 and SEMG2. This indicated that BA improved the infertility of rats. In general, BA might alleviate depression and infertility in CUMS + LPS rats through the *β*-catenin signaling pathway.

The previous study has shown that BA could alleviate depressive behavior in CUMS-induced mice and protect nerves [44]. Therefore, we also tested the depressive behavior of CUMS + LPS rats. Our results indicated that BA significantly increased the levels of SPT and OFT in rats and reduced the levels of TST and FST. The above results revealed that BA reduced the depression-like behavior of rats. The higher the concentration of BA, the more obvious the effect.

To explore the effect of BA on the infertility symptoms of CUMS + LPS rats, we tested the fertility of rats. The results showed that the fertility of CUMS + LPS rats was significantly reduced. After the BA treatment, the reproductive ability of rats was improved. A semen sample with poor sperm motility was called asthenospermia, which was considered to be one of the main factors leading to male infertility [47]. In this study, the sperm motility of CUMS + LPS rats was decreased notably. After BA treatment, the sperm motility has been improved. All the results showed that CUMS + LPS caused the infertility of rats, while BA treated the infertility symptoms.

## 5. Conclusion

BA attenuated LPS-induced DCMI by regulating *β*-catenin expression. In addition, our results for the first time demonstrated the potential mechanism by which BA regulated the expression of *β*-catenin in CUMS rats.

## Data Availability

The data used to support the findings of this study are available from the corresponding author upon request.
